# Language of Religion, Religions as Languages. Introduction to the Special Issue ‘Religions and Languages: A Polyphony of Faiths’

**DOI:** 10.1007/s11841-022-00912-5

**Published:** 2022-03-31

**Authors:** Andrea Vestrucci

**Affiliations:** 1 Graduate Theological Union, Berkeley, CA USA; 2 University of Geneva, Geneva, Switzerland

**Keywords:** Language of religion, Religion as language, Interreligious dialogue, Religion and technology, Religion and arts, Religion and science

## Abstract

Religions use linguistic and non-linguistic codes of meaning to express their contents: natural tongues, music, sculpture, poetry, rituals, practices... Also, religions provide the semantic context and the rules to produce, validate, and interpret their expressions: as such, religions can be considered languages. The *Sophia* Special Issue ‘Religions and Languages: A Polyphony of Faiths’ explores the multifaceted relationships of world religions with languages broadly construed, intended as other religious codes, natural tongues, artistic forms, digital media, and even science. Do natural languages modify themselves in order to convey a divine message? How do artistic means of expression accommodate religious contents? What are the aspects of interaction between religions, technological advances, and scientific methods? The five contributions in this issue offer innovative, compelling, and engaging perspectives regarding this complex and fascinating issue.

We do not choose our family. We do not choose our name. Along with our family and our name, we also do not choose the natural language(s) we learn during the first years of our life—our ‘mother tongue(s).’ Mother tongues play an essential role in our interaction with the world and in our orientation in the world. Every aspect of our world experience passes through language—even if it is just to say: ‘Words fail me!’ Thus, language modifies the world: our world is a world that is *said* in a specific tongue.

Since there are many natural languages, perhaps different languages enable different access to the world. Are there as many relationships to the world as languages? Is the so-called Sapir-Whorf Hypothesis true? Perhaps, one of the problems in demonstrating that hypothesis is precisely language’s omnipresence: language is simultaneously the means used to demonstrate the hypothesis and the object under examination.

Another thing we might receive at the beginning of our life is a religious identity. The reception of this identity comes in at least two ways: a direct way and an indirect way. By ‘direct way’ I mean the presentation (more or less free) of a religious identity by the family group or religious order (e.g. baptism). By ‘indirect way’ I mean the religious presence that marks the area, town, or country in which we grow.

In light of this, one can infer that a religious message is claimed to be true only for the habitude of being imbued with a religious tradition—in most cases the tradition of the family or of the birth/living place. The constant exposure to this religious tradition transforms a religious message into an unquestionable truth. So—the argument goes—the supposed truth of a religious message is a matter of self-delusion because it does not depend on the message itself but just on the reiterated and/or coercive contact with the message (Dawkins, 2016).

I make two observations on that. First, a religion can surely have a core set of ‘truths’ (messages, teachings, doctrines) that circumscribe the identity of this religion and its difference from the others, but this core set is not necessarily consistent (it is not a set of axioms), and for this reason, it is open to reinterpretations, rearrangements, and diversifications. Otherwise, there would be no history of religions, but just a crystallized and disembodied doctrinal recapitulation. Second, religious truths are universal in a very peculiar way: they activate only within the specific religion in which these truths are formulated. In other words, the validity of a religion’s truths spans across all possible expressions of that religion (rituals, discourses, practices…), precisely because these truths establish the connection between these expressions and the actual existence of that religion; therefore, the validity of a religion’s truths does not span across the expressions of other religions.^1^ I will develop this shortly.

The same argument can be applied to anything uttered in any natural language, and not only religious ‘truths.’ In light of the plurality of natural languages, the specific access to the world provided by a language *x* might not be understood in a language *y*. Different hermeneutics are in place in different linguistic settings.

However, for natural languages, there is *translatability*: something expressed in a language can be translated into another language with no (remarkable) semantic loss—perhaps with the exception of poetry.^2^ ‘The snow is white,’ ‘बर्फ सफेद है,’ ‘雪は白い,’ A hó fehér’ … the same message can be formulated in different languages.

Between religions, translatability is more complex. Sure, the teachings of a religion can be translated (more or less successfully) into multiple languages—the evidence of this is that some religions are practiced in very different tongues. But translatability from one religion to another is a challenge, because it is hard to find a common semantic ground between religions. Translating ‘The snow is white’ across languages is easy because there is a common semantic ground: the shared empirical experience that the object called ‘snow’ has the property of being ‘white.’ In sum, this common ground is the definition of an object of common experience. In the case of religions, translating a religious expression across religions is difficult, because the ‘object’ defined, represented, or conveyed by this expression (one or more deities, a notion, a virtue…) is not common, but it is specific to this or that religion—and it is specific because it characterizes this or that religion and its difference from other religions. Thus, the semantic commonality between religions is not linear.^3^

This semantic non-linearity across religions is just the tip of the iceberg. Religions provide not only their symbols’ meanings (the semantics), but also the ways of combining such symbols (the syntax), the definition of the context for these symbol combinations (the pragmatics), and in some cases even the symbols’ sacred origin (a sort of semiotics). In sum, religions provide the rules of composition (the grammar) and of validation (the logic) of their own expressions—either explicitly, e.g., in a set of core teachings (sacred texts, tenets, dogmas …), or implicitly, e.g., in living practices and habits. Hence, the semantic encounter between religions is not linear because it is also an encounter between specific (religious) grammars and logics, i.e., between definitions of specific (religious) linearities. In other words, religious truths are ‘universal’ in the sense that they are valid within, and *only* within, the semantic, syntactic, and pragmatic contexts defined by a specific religious grammar and logic. It follows that each religion is not just a specific use of language: it is a specific language on its own—with vernaculars, neologisms, contradictions, and developments. As it makes no sense to say that a natural language is superior to another language, so it makes no sense to say that a religion is superior to another religion.

The issue of translatability is further complicated by the notion of sacred language: many religions attribute to a specific language a holy status, as the elective vessel to convey a divine message. Qur'anic Arabic, L’shon Hakodesk, Sanskrit, and Classic Armenian are just a few examples of sacred language. Any translation from a holy linguistic vessel into a ‘secular’ natural language loses some of the sacrality of what is stated in that sacred language. Thus, religions are multi-layered, internally complexified systems of communication.

These reflections invite the formulation of the hypothesis that religions use natural languages not only to convey a message *through* these languages, but also to convey a religious message *about* these languages: the message that no human language is able to fully express a divine message. Thus, religions might use language ‘against itself,’ so to say, in order to formulate the idea that, from a religious perspective, all human languages are limited. When used to express a religious meaning, a language simultaneously expresses *its own* religious limitation (Vestrucci, 2019).

However, religions do not use only natural languages, but also other ‘languages’ *lato sensu*, i.e., other codes of meaning, other systems of human communication. One of these codes is arts. It is difficult to find a religion which does not use music and rhythms in rites, meditations, holy readings, etc. Moreover, architecture, sculpture, abstract or figurative decorations, and various techniques of painting are all employed in religious settings. Another example is poetry, or, more generally, the metaphoric use of language; thanks to its rhythmic component, poetry is used in sacred texts, ritualistic formulae, and prayers.

Also, religion interacts with other specific and widespread codes such as the languages of technology and of science. There are negative and positive examples of such interaction: the recent COVID-19 pandemic has forced the invention of alternative, technology-driven forms of worship and spiritual care; religions might refer to scientific explanations both as challenges (as in the case of cosmological and evolutionistic theories) and as resources (as in the case of the ecological emergence inviting to reformulate interreligious responsibility) (Sherma & Bilimoria, 2022).

The *Sophia* Special Issue ‘Religions and Languages: A Polyphony of Faiths’ explores the multifaceted relationship of world religions with languages *qua* codes of meaning. These codes can be other religious languages, and in this case, the relationship is a (more or less harmonic) polyphony of faiths. Or these codes can be natural languages, artistic codes, digital media, and science. Do natural languages modify themselves in order to convey a divine message? How do artistic forms of expression accommodate religious contents? What are the different aspects of interactions between religions, technological advances, and scientific methods? The five contributions in this issue offer highly innovative, compelling, and engaging perspectives in this complex and fascinating issue.

The first contribution, by Francis X. Clooney, presents an encounter between two religions, Hinduism and Christianity, in two specific forms: Vaishnavism and Catholicism. This encounter is built in two moments: the first moment is the contact made by Clooney, a highly respected academic and a Jesuit, with the verses of *Tiruviruttam*, a sacred poem written by the great Tamil mystic poet Śaṭakōpan (eighth century C.E.); the second moment is the comparison between a verse of this sacred Hindu text, and a verse of the *Song of Songs*, one of the books constituting the sacred text of Judaism and Christianity. The connection between the two moments is the attitude of the reader—Clooney, and we alongside with him: the attitude of the pilgrim. A pilgrim leaves the motherland to begin a journey in distant lands, entering in contact with people of different rites, different languages, and different texts. The pilgrim approaches this foreign reality respectfully because this reality contains a glimpse of what constitutes the pilgrim’s own identity: after all, beneath the specific differences, the pilgrim still finds a(nother) language, (other) rites, and (other) texts. This contact allows the pilgrim to formulate new insights on their own language, rites, and sacred texts, and thus to deepen their own religious identity. This is precisely what happens in this article: the accurate analysis of the verse from *Tiruviruttam* opens to rediscover the role of poetry in sacred texts as expression of the limit of human language in conveying a divine message.

The second contribution, by anthropologist Lionel Obadia, studies the cross-contaminations between two apparently distant codes: magic and technological development. On one hand, we have magic, something that is usually considered, perhaps too hastily and superficially, a form of thinking which is connected with irrationality and superstition. On the other hand, we have technology and its development, something that is usually considered and praised as the ultimate achievement of human intelligence and as the most evident mark of the progress of humankind. By engaging these ‘usual considerations’ and our *prima facie* approaches to magic and technology, Obadia’s article reveals hidden affinities between the two codes and unveils their unexpected facets, thus helping us recalibrate our approach to them. The article analyzes different layers of the relationship between magic and technology: magic is a form of technology in the meaning of a ‘way to modify the world,’ and technology seems to perform things magically, i.e., in a way that defies all reasonable explanations; where present, both magical and technological attitudes permeate human life, and both express complex symbol-manipulation. The article focuses specifically on Artificial Intelligence (AI): it underlines how AI developments serve human ‘magical’ need to transcend our finite condition, and it synthesizes the religious metaphors used in AI-related dystopias.

​The third contribution focuses on the interconnections between three languages: geometry, fine arts, and religion. In her article, Islamic art historian Wendy Shaw explores Islam’s artistic representation of the divine through non-figurative, geometric artistic means. She hints at the religious interpretation of a psychological disposition to recognize an affinity between geometric order and divine order. From this, Shaw underlines the non-semiotic character of geometrical patterns in Islamic arts: the meaning that geometrical arts convey are particular to non-figurative representations. Thus, in contrast to many nineteenth century European considerations, geometrical patterns do not constitute a grammar, but a potential spiritual message. The contribution reconstructs the changes in European interpretations of Islamic geometrical arts, by underlying the shift from an ‘etic’ concept of arts to an ‘emic’ approach focusing on the artistic expression of religion in several cultural frameworks. Islamic geometric art contributes to recalibrating the tension between the secular and the sacred: the imitative expression of the divine in geometric patterns makes it possible to retranslate a spiritual meaning into the observer’s semantic coordinates. The contribution applies this non-semiotic interpretation of geometrical patterns to modern art by presenting a comparative analysis between the works of Iranian artist Shahroudy Farmanfarmaian and Dutch artist M. C. Escher. In both artists’ works, geometrical forms are not semantic placeholders: the meaning they express coincides with ideas suggested in Islamic sources, even though they are not overtly or intentionally religious.

Fourth, philosopher of science and theologian Yiftach Fehige leads us to explore the interactions and distinctions between science and religion, specifically the natural sciences and Judeo-Christian religious tradition. Fehige focuses on the distinction between language and imagination to explain the complex equilibrium between these two different expressions of human intelligence. As the contribution’s title synthesizes, Fehige entertains the notion that science and religion are separated by their specific languages, but they are united by the common use of imagination as the heuristic tool to guide, on one hand, scientific research and, on the other hand, theological speculation. Imagination is at the basis of creativity, which activates when one needs to conceive an empirical or thought experiment to confirm a scientific theory, to express hidden affinities in fine arts, or to formulate intuitions about the divine in theology. Thus, imagination plays a fundamental role in the investigation of truth in science, arts, and religion, providing unity between them. In support of this idea, Fehige refers to the analysis of imagination in the writings of mathematician Jacob Boronsky, philosopher of science Menachem Fisch, physicist Thomas McLeish, and theologian David Brown. Particularly evocative is Fehige’s analysis of the story of the Tower of Babel, where Old Testament exegesis meets epistemological and methodological reflections to unveil unexpected complexities in the Biblical story.

The last contribution, by scholar of religion Andrea Rota, analyzes the speech acts characterizing a specific component of religion: ritual practices. Rota harks back to the notion of ‘collective intentionality’: in specific circumstances, a group of individuals acts, thinks, and/or feels in a way that reflects the intentions of the group itself, rather than of its individual members. Religious rites may explain the emergence of such circumstances. The article focuses on the linguistic structure of Jehovah’s Witnesses’ congregational practices, and in particular the question-and-answer section of JW worship. Rota underlines that, in this dialogical section of the worship, it is a common practice to limit the use of the first and second singular pronouns in favor of the first plural pronoun. The emphasis on the ‘we’-dimension determines a collective intentionality which, at its turn, is expressed through the joint commitment towards active ministry for the congregation’s sake. This explains the apparent contradiction in empirical data collected by Rota between, on one hand, the collective agreement to perform a ministry action (e.g., door-to-door ministry) and, on the other hand, the uneasiness to perform the same action whenever conceived as an individual act. As such, the contribution provides pioneering evidence for the fruitfulness of a real interdisciplinary interaction between analytical philosophy and the social sciences, via the mediation of speech act theory.

## Footnotes

1 This begs the question about how it is possible to group religions under a common label unless there is a basic religious structure or even a universal religious grammar of which all religion are specifications. Pure theoretical models risk to be disembodied from living practices, and empirical studies need further development (Oviedo & Canteras, 2013).

2 The more we shift from world descriptions to abstract notions, the more translation might become problematic as there might not be a 1 : 1 correlation between source language and target language (see Van Orman Quine, 2013: 70). However, this does not negate translatability: it rather expands translatability to a 1 : (*x* > 1) correlation between source language and target language—i.e., it simply confirms that translation is always possible *and* improvable (see Davidson, 1973: 19). Poetry is a good example of the limitation of translatability because it uses figures of speech and word organizations that defy the semantic and syntactic boundaries of the specific natural language in which poetry is composed. Yet, poetry does not negate translatability because poetry is still part of the expressions of that natural language, and as such it is translated (more or less successfully). In sum, there is no ‘neutral language’ from which ‘objectively’ evaluating translations and/or claiming that no translation is possible between two languages; there are just *specific* languages, and we evaluate translation from within this or that specific language which translates, and which is translated. After all, to speak means always *already* to be in the act of translating.

3 Even the (common? shared?) experience of a connection with an ‘outer’ dimension (a mystical/meditative status of rapture, pristine gratitude, unconditioned forgiveness, deep unity, and/or awe) is triggered, lived, and communicated through codes, rites, practices, notions, and representations that are specific to each religion.
